# Moving Away from One-Size-Fits-All: Assessing the Use of Pharmacogenetic-Guided Medication Therapy in Pediatric Patients with Chronic Pain

**DOI:** 10.3390/children12060721

**Published:** 2025-05-31

**Authors:** Danielle Ruskin, Klaudia Szczech, Sierra Scodellaro, Naiyi Sun, Iris Cohn

**Affiliations:** 1 Department of Psychology, The Hospital for Sick Children, Toronto, ON M5G 1X8, Canada; klaudia.szczech@unityhealth.to; 2 Department of Anesthesia and Pain Medicine, The Hospital for Sick Children, Toronto, ON M5G 1X8, Canada; naiyi.sun@sickkids.ca; 3 Department of Psychology, York University, Toronto, ON M3J 1P3, Canada; 4 Women’s and Children’s Health Program, St. Michael’s Hospital, Unity Health Toronto, Toronto, ON M5C 2T2, Canada; 5 Division of Clinical Pharmacology and Toxicology, The Hospital for Sick Children, Toronto, ON M5G 1X8, Canada; sierra.scodellaro@sickkids.ca (S.S.); iris.cohn@sickkids.ca (I.C.); 6 Department of Anesthesiology and Pain Medicine, Temerty Faculty of Medicine, University of Toronto, Toronto, ON M5S 1A8, Canada; 7 Department of Pediatrics, Temerty Faculty of Medicine, University of Toronto, Toronto, ON M5S 1A8, Canada

**Keywords:** pediatric chronic pain, chronic pain service, pharmacogenetics (PGx), precision medicine, qualitative analysis

## Abstract

**Background/Objectives:** Pharmacogenetic (PGx) testing can predict drug efficacy, toxicity, and risk of adverse drug reactions (ADRs). However, PGx-guided prescribing for pediatric chronic pain is underutilized. **Methods**: We evaluated the rate of deviance from standard drug dosing regimens in children and adolescents with chronic pain based on PGx testing of drug-metabolizing genes. We also assessed the acceptability and feasibility of PGx testing and implementation of PGx-guided recommendations from patient, caregiver, and prescriber perspectives. Finally, we explored whether PGx results could predict self-reported therapeutic responses and/or ADRs to medications. **Results**: Forty-eight participants aged 8–17 years with chronic pain provided DNA via buccal swab. Genetic variant data for *CYP2D6*, *CYP2C9*, and *CYP2C19* metabolism genes and associated metabolizer status were analyzed with respect to clinical PGx guidelines for dosing recommendations of analgesics and psychotropic medications. Participants, their caregivers, and their prescribers also completed quantitative questionnaires evaluating their experience with PGx testing. Twenty-three (50%) participants were predicted to benefit from non-standard dosing for medications with clinical PGx guidelines. Participants expressed satisfaction with the PGx testing process and felt it was safe and worthwhile. Prescribers also reported that PGx results were relevant for medication choices in 42 (91%) participants. Seven (15%) participants had genotyping results which may have predicted their self-reported therapeutic responses and/or ADRs to specific medications. **Conclusions**: Though further research on pharmacodynamic associations is required to sufficiently address the complexity of interpatient responses to medications for the treatment of pediatric pain and mental health conditions, PGx testing may be used to inform individualized medication choices based on genetic make-up.

## 1. Introduction

Pediatric chronic pain is highly prevalent, undervalued, and undertreated [1,2,3]. One in four pediatric patients experience persistent pain where analgesic medications are part of pain treatment regimens [4]. Given the well-documented co-occurrence of mood and anxiety disorders among children and adolescents with chronic pain [5], a portion of these patients presenting to pediatric pain clinics are already likely taking or are recommended to take a psychotropic medication for a primary psychiatric disorder. Comorbid psychiatric symptoms can impact children’s and adolescents’ presentations at pain clinics by exacerbating pain symptoms, contributing to functional impairment and discomfort, and reducing overall quality of life [6]. Additionally, children and adolescents prescribed analgesic medications and psychotropic drugs are at increased risk of developing adverse drug reactions (ADRs) [7,8,9,10]. Frequent cycles of trial and error are common when finding the most effective medication and dose to manage pain and mental health [11]. With significant interpatient variability in both pain perception and pain reduction after treatment, a one-size-fits-all approach to pain management in pediatric patients is often not sufficient [12].

Pharmacogenetic (PGx)-guided medication therapy is the low-hanging fruit of precision medicine and incorporates differences in genetic make-up to guide medication selection and dosing for patients [13,14]. PGx-guided medication prescribing is already available via published clinical practice guidelines created by various professional entities, including the Clinical Pharmacogenetics Implementation Consortium (CPIC) [15]. Guidelines exist for many commonly prescribed analgesics, such as ibuprofen, celecoxib, tramadol, and codeine, as well as commonly used antidepressants for pain, with the aim of reducing ADRs and improving the overall therapeutic response. Genetic variation in medication-metabolizing genes, such as *CYP2D6*, *CYP2C9,* and *CYP2C19*, can influence blood drug levels, which may contribute to either the risk of ADRs or therapeutic failure to standard dosing [16]. Various studies have summarized the current impact of PGx on opioid metabolism, as well as nonsteroidal anti-inflammatory drugs (NSAIDs) and antidepressants, as they are also important pillars to current treatment strategies of pain [17,18,19]. Although much of our knowledge regarding PGx and its impact on medication safety and efficacy in controlling pain is limited and controversial, reports have demonstrated promise in improving therapeutic outcomes [20,21,22].

The primary objective of this study was to determine the rate of deviance from standard pain management and psychotropic medication dosing regimens in pediatric patients with chronic pain based on PGx testing results. A secondary objective involved evaluating the feasibility, acceptability, and implementation of PGx testing and dosing recommendations from the perspectives of patients, caregivers, and their health care providers (HCPs) who prescribe analgesics. An exploratory objective was to determine whether PGx results could predict individual responses to medications based on self-reported medication use and ADRs.

## 2. Materials and Methods

### 2.1. Study Design

This preliminary multidisciplinary pilot study utilized a prospective non-randomized cohort design to assess the acceptability, feasibility, implementation, and clinical utility of the PGx-guided prescribing of analgesics in a small cohort of children and adolescents with chronic pain conditions. As a pilot study, this work was exploratory in nature and not designed to test formal hypotheses but rather to inform the design and methodology of future hypothesis-driven research [23].

### 2.2. Participants

A convenience sample of 48 pediatric patients and their caregivers was recruited between May 2022 and August 2023. Sample size was chosen based on the average number of referrals to the chronic pain clinic (CPC) per year and suggestions for internal pilot studies [23,24]. Patients were eligible to participate if they were between 6 and 18 years old, could speak and read English, and were referred for care to the CPC at the hospital where the research took place. Please see Ruskin et al. [25] for a description of the multidisciplinary pediatric CPC intake assessment that all patients receive and where pharmacological recommendations are made. Pain diagnoses were made by a pain physician during the intake visit based on clinical assessment and documented in the medical record (see Table 1 footnote for details). Chronic pain was defined and categorized according to the ICD-11 classification system [26]. Psychological diagnoses were documented in psychologist notes and confirmed via standardized psychological diagnostic assessment. Patients were excluded if they required language interpretation services or cancelled their CPC referral.

### 2.3. Setting

The current study was conducted in an outpatient chronic pain clinic at a large urban pediatric tertiary care hospital. Ethics approval for this research was obtained from the institution’s Research Ethics Board (REB #1000053445). Informed consent, including assent and parent/guardian consent, was obtained from all subjects involved in the study.

### 2.4. Recruitment

Following institutional research ethics approval, patient medical charts were reviewed for eligibility, and those meeting eligibility criteria were introduced to the study by a member of their circle of care. Participants were recruited via two pathways: (1) patients on the CPC waitlist were offered preemptive PGx testing in advance of their first appointment, and (2) patients were referred for reactive PGx testing by their physician at an existing CPC appointment. Interested patients and caregivers were then approached by a member of the research team (KS, SS) to obtain informed consent using an online consent form through Research Electronic Data Capture (REDCap), a secure web-based application designed to support data capture for research studies [27].

### 2.5. Data Collection

Enrolled participants were mailed sample collection kits to their home address and administered the Medication Use History Questionnaire (see Appendix A, Table A1) via email using a REDCap weblink. Participants completed at-home buccal swab sample collection and returned the completed kit with provided postage to a Clinical Laboratory Improvement Act (CLIA)-certified PGx testing facility. Samples were analyzed, and data were composed into a PGx report as described in detail below (Section 2.6 and Section PGx Report). The report was then added to the participant’s electronic medical record (EMR). In addition, a consultation was scheduled with members of the research team specialized in PGx (IC, SS) during which the report was reviewed with participants and their caregivers. Consultations were approximately 1 h and delivered via virtual care (PHIPA [Personal Health Information Protection Act]-compliant Zoom for Healthcare). Patient and caregiver participants completed the PGx Testing Acceptability Questionnaire (Appendix B, Table A2) by REDCap weblink one day after their consultation. The PGx report was also shared with the participant’s care team (i.e., primary care physician, referring provider, pharmacist, as applicable) by secure internal email immediately after the consultation and again in advance of their CPC appointment when medications are prescribed (point of care [POC] visit). Prescribers completed the PGx Testing Implementation/Acceptability Questionnaire (Appendix C, Table A3) by REDCap weblink following each participant’s POC visit. Families were emailed a REDCap weblink to the PGx Testing Follow-up Questionnaire (Appendix D, Table A4) at a 3-month follow-up after their POC visit. Finally, all HCPs who had a patient enrolled in the study were emailed a REDCap weblink to the PGx Testing Overall Feedback Questionnaire (Appendix E, Table A5) once study recruitment was complete. All quantitative data collection measures were distributed via email and stored on REDCap.

### 2.6. PGx Testing Methods

One self-collected buccal swab was obtained from each patient participant and mailed to a CLIA-certified PGx testing facility. Genotyping of *CYP2C19*, *CYP2C9*, *CYP2D6*, *CYP3A5*, *F5*, *NUDT15*, *SLCO1B1*, and *VKORC1* was carried out using the Agena MassARRAY^®^ platform. Descriptive data analysis was limited to variants in *CYP2C19*, *CYP2C9,* and *CYP2D6* given their association to treatment response for various psychotropic and pain medications (Appendix F, Table A6). DNA samples were normalized to a concentration of 10 ng/µL, and 2 µL per well was used for PCR amplification and primer extension with the iPLEX, iPLEX Veridose Core, and Veridose *CYP2D6* CNV reagents (Agena Biosciences, San Diego, CA, USA). Thermal cycler Biorad C1000 (Bio-Rad Laboratories, Hercules, CA, USA) was used for amplification. The extension products were dispensed onto a CPM 384 SpectroCHIP Array (Agena Biosciences, San Diego, CA, USA) using the Agena 384 (Agena Biosciences, San Diego, CA, USA) chip prep module and detected using a MassARRAY MALDI-TOF mass spectrometer (Agena Biosciences, San Diego, CA, USA), providing genotyping and quantification. Haplotype reports were automatically generated using the Typer software (v5.0.2) and the ADME PGx Pro software (v3.99.10, Agena Biosciences, San Diego, CA, USA), according to the manufacturer’s standard protocols. Results were processed to generate single-nucleotide polymorphism (SNP) calls automatically using the MassARRAY^®^ TyperAnalyzer software (Agena Biosciences, San Diego, CA, USA), and then manually reviewed by the operator to validate the allele calls. Automatic SNP calls that were of concern were removed.

#### PGx Report

Participants’ genetic variant data, including variants in the *CYP2D6*, *CYP2C9*, and *CYP2C19* metabolism genes and associated metabolizer status, were collected from a gen-otyping record generated by a CLIA-certified laboratory. The record was reviewed, inter-preted, and composed into a report by members of the research team specialized in PGx (IC, SS). Only clinically recognized genetic variants known to affect response to drug therapy or risk of ADRs were reported. These variants were identified by comparing the participant’s variants against those in the PharmGKB database, a comprehensive data-base for significant pharmacogenomic findings [28], and the FDA Table of Phar-macogenomic Biomarkers in Drug Labelling which details clinically actionable drug–gene combinations [29].

The targeted genotyping technology used in this study is only able to inform about variants available on the test panel. The composed PGx report details specific dosing recommendations, along with additional supporting drug–gene information that may help guide current and future medication prescribing. Throughout this manuscript, the term “PGx medication” will be used to denote medications with PGx prescribing guidelines listed in the PGx report. A copy of the PGx report with all tested genetic variants was provided to participating families and their primary care team to ensure the PGx report was considered for future clinical management.

### 2.7. Measures

#### 2.7.1. Demographics

Demographic data, including age, sex, pain diagnosis, psychological and concurrent diagnoses, use of pharmacological and non-pharmacological treatment (i.e., physiotherapy, psychiatry, psychology, occupational therapy), and pain characteristics (duration, interference), were collected from participants’ medical charts. Demographic data were collected from pediatric participants (not caregivers). Feasibility and satisfaction data were collected from both children and caregivers. The following self-report measures were developed for the purposes of this preliminary investigation with consensus from all authors of this study, and as such, are not standardized psychometric instruments and do not have established psychometric properties.

#### 2.7.2. Medication Use History Questionnaire

The Medication Use History Questionnaire was administered at baseline to gather participants’ medication use history, including medications taken prior to study enrollment, history of ADRs and therapeutic efficacy, and previous prescribers of medication.

#### 2.7.3. PGx Testing Implementation/Acceptability Questionnaire—Prescriber

The PGx Testing Implementation/Acceptability Questionnaire for prescribers asked whether HCPs prescribed a medication at the POC visit, whether it was a medication of interest that carries PGx implementation guidelines, and whether any available PGx testing recommendations were implemented.

#### 2.7.4. PGx Testing Follow-Up Questionnaire—Patient and Caregiver

The PGx Testing Follow-up Questionnaire for patient and caregiver participants included questions about participants’ medication use since being enrolled in the study, any side effects and reports of efficacy, who prescribed, whether the prescriber used the PGx testing results, and whether PGx testing improved confidence and willingness in taking medication. Definitions for symptom change were derived from the Patient Global Impression of Change Scale [30] and are listed in the note under Table 5, as well as in Appendix D, where the questionnaire is provided.

#### 2.7.5. PGx Testing Overall Feedback Questionnaire—Prescriber

The PGx Testing Overall Feedback Questionnaire for prescribers queried their satisfaction with the PGx testing process and overall implementation of PGx testing results.

### 2.8. Analytical and Statistical Approach

Descriptive data were computed to assess participant characteristics, medication usage, the metabolizer status of CYP2C9, CYP2C19, and CYP2D6 drug-metabolizing enzymes, ADRs, and feasibility and acceptability data. The rate of deviance from standard prescribing of analgesics and psychiatric medications listed on the PGx report was determined by the proportion of participants carrying a genetic variant associated with PGx prescribing guidelines. Feasibility was assessed by (a) the proportion of participants who received PGx results and recommendations prior to their POC visit for prescription of an analgesic and (b) the proportion of patients approached who consented to PGx testing. Acceptability was assessed by (a) the perceived utility of PGx test results for current and future pharmacological pain management decisions from patient, caregiver, and HCP perspectives; (b) participants’ understanding of PGx results, reports, and consultations; and (c) whether PGx testing increased participants’ confidence and willingness to take medication. Implementation was assessed by the proportion of cases where HCPs implemented PGx testing recommendations. To assess our exploratory objective, we determined the proportion of participants with genotyping results which corresponded to (i.e., may have predicted) their self-reported therapeutic responses and/or presence of ADRs in the Medication Use History Questionnaire.

## 3. Results

### 3.1. Recruitment and Retention

**Figure 1:**
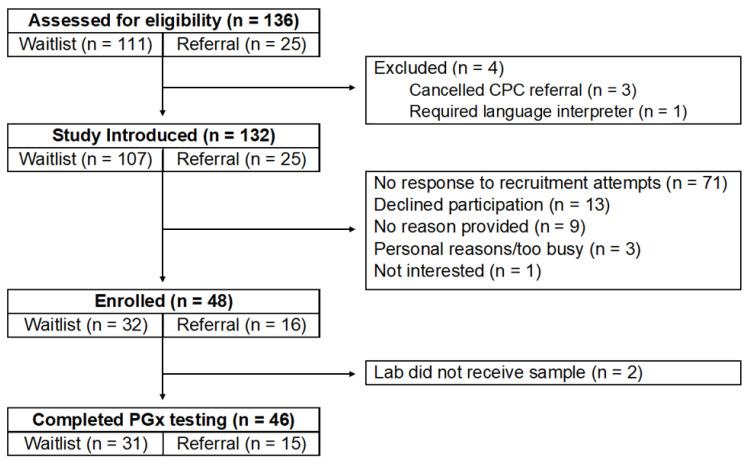
Study accrual by recruitment pathway.

Of the 136 patients screened, 132 (97%) were eligible and offered PGx testing. Of those eligible, 48 (36%) agreed to participate and 46 (35%) completed testing (Figure 1).

### 3.2. Participant Characteristics

Means, frequencies, and standard deviations were computed for demographic data across participants for age, sex, pain diagnosis and duration, other concurrent diagnoses, and psychosocial variables (Table 1). Participants were primarily female (35; 76%), had an average pain duration of 3.6 years, and 33 (72%) participants had at least one concurrent psychiatric diagnosis. On standardized questionnaires, participants reported elevated levels of pain interference, anxiety, depression, and pain catastrophizing, along with below-average mobility ratings.

### 3.3. Medication Usage

At study enrollment, 29 (63%) participants were already taking at least one analgesic (25/29; 86%) and/or psychotropic medication (13/29; 44%), including but not limited to acetaminophen, morphine, gabapentin, naproxen, duloxetine, and fluoxetine. Among the entire cohort, 24 (52%) participants were taking a medication that carries published clinical PGx practice guidelines at the time of their report being available. Of those, 13 (54%) were taking a PGx-listed analgesic, while 12 (50%) were taking a PGx-listed psychotropic medication (Table 2).

### 3.4. Drug-Metabolizing Enzyme Status

**Figure 2:**
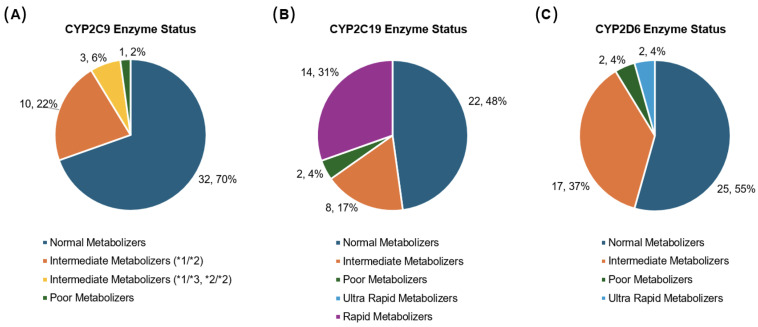
(**A**) CYP2C9, (**B**) CYP2C19, and (**C**) CYP2D6 metabolizer statuses among study cohort, N = 46. These proportions correlate with expected population allele frequencies.

Many of the commonly observed PGx medications in this cohort are metabolized by the following enzymes: CYP2C9, CYP2D6, and CYP2C19. Figure 2 summarizes the distribution of metabolizer status for each enzyme determined by participants’ PGx test results. One participant (2%) was classified as a CYP2C9 poor metabolizer (i.e., carrying two no-function alleles, such as * 2/* 3 or * 3/* 3) while three (7%) were predicted to be intermediate metabolizers (i.e., carrying one normal-function allele plus one no-function allele or two decreased-function alleles, such as * 1/* 3 or * 2/* 2) potentially affecting their response to NSAIDs by increasing the probability and severity of toxicities [31].

Two (4%) participants who were predicted to be CYP2C19 poor metabolizers, as well as 14 (30%) participants predicted to be CYP2C19 rapid metabolizers, would have benefitted from altered SSRI dosing as per the relevant CPIC guidelines [32]. Additionally, CYP2C19 rapid metabolizers who were prescribed first-generation proton pump inhibitors (PPIs), such as omeprazole for acid reflux treatment and other gastrointestinal disorders, may have experienced impaired PPI efficacy due to increased clearance of the drug, according to the respective CPIC guideline [33]. Collectively, 16 (34%) participants are predicted to benefit from non-standard dosing of SSRIs or PPIs based on their CYP2C19 enzyme metabolizer status.

For CYP2D6 enzyme function, two (4%) participants were predicted to be poor metabolizers and two (4%) were ultra-rapid metabolizers, impacting opioid use following CPIC recommendations. In such cases, alternatives are suggested over codeine or tramadol, given the potential for reduced analgesia or an increased risk of serious toxicities, respectively [34].

### 3.5. Primary Objective

#### Rate of Deviance from Standard Dosing Regimens Based on PGx Testing Results

Twenty-four (52%) participants were taking at least one medication with clinical PGx guidance as part of their pharmacotherapy regimens at the time they received their PGx test results. Based on genotyping results, five (21%) of these participants were predicted to benefit from non-standard dosing of their current medication treatment. This immediate guidance was related to the use of ibuprofen and SSRIs based on the CYP2C9 and CYP2C19 genotypes, respectively. Overall, 23 (50%) participants were predicted to benefit from non-standard dosing for pain and psychotropic medications, such as NSAIDs, opioids, SSRIs, and SNRIs, that carry clinical PGx guidelines.

### 3.6. Secondary Objective

#### 3.6.1. Feasibility of PGx Testing

The rate of study enrollment was higher for participants approached during their POC visits by a clinical team member (i.e., referral group), with 16 (60%) enrolled of 25 approached, compared to those approached while on the waitlist (32 (30%) enrolled of 107 approached). All participants in the referral group had their PGx reports uploaded to their EMR and received their PGx consultation prior to their POC visit with the healthcare team. However, 23 (74%) participants in the waitlist group had their PGx report uploaded into their EMR prior to their POC visit, and 18 (58%) received their PGx consultation prior to the POC visit (Table 3). In several cases, PGx results were not available due to factors unrelated to the PGx testing process, including participant delays in submitting samples and POC visits being rescheduled to earlier dates.

#### 3.6.2. Acceptability of PGx Testing

Acceptability of PGx testing was generally very high. Of the participants who completed study questionnaires (38 patients and 40 caregivers), 36 (95%) patients and 39 (98%) caregivers agreed that PGx testing was worthwhile, safe, and had reassured them that their healthcare team was selecting the best medication for them/their child. A total of 34 (89%) patients and 34 (85%) caregivers agreed that PGx testing reduced uncertainty about their/their child’s response to medications, while 31 (82%) patients and 38 (95%) caregivers agreed that PGx testing increased their confidence in the effectiveness of medication for pain control (Table 4).

#### 3.6.3. Implementation of PGx Testing

Based on results from the HCP Implementation/Acceptability Questionnaire, HCPs reported that PGx testing results were relevant for past, current, and/or future medication choices in 42 (91%) participants. In the seven cases where participants’ PGx reports were available at the time of the POC visit and HCPs prescribed a medication with PGx guidelines, they implemented PGx recommendations in all but one case (83%).

### 3.7. Exploratory Objective

#### Predicting Therapeutic Response Based on PGx Results

Self-reported lifetime medication use, along with therapeutic response and/or ADRs, was collected via the Medication Use History Questionnaire (Appendix A, Table A1). These data were analyzed in combination with participant PGx test results to determine whether the PGx result could have informed individual responses to medications that carry PGx-guided prescribing recommendations. Of all reported responses, PGx test results may have been predictive for seven (15%) participants (Table 5). One participant noted an improvement in symptoms while taking sertraline and ondansetron, of which their normal CYP2C19 and CYP2D6 metabolizer status, respectively, may corroborate. Two participants taking lansoprazole reported moderate improvement to symptoms. They were both categorized as CYP2C19 intermediate metabolizers, thus potentially able to experience an increased probability of efficacy given elevated levels of active drug. Three participants, one CYP2D6 poor metabolizer and two CYP2C19 rapid metabolizers, reported no change in symptoms after taking either codeine, pantoprazole, or citalopram, respectively. These metabolizer statuses confer reduced clinical effect of these medications, potentially explaining the lack of therapeutic benefit experienced. Finally, one participant categorized as an ultra-rapid CYP2D6 metabolizer reported side effects to codeine, possibly explained by their PGx findings, which predict an increased probability of adverse effects.

## 4. Discussion

Analgesics and psychotropic medications are standard components of pediatric chronic pain treatment programs. However, these medications are often prescribed without knowledge of a patient’s PGx metabolizer status, which may influence their response to standard drug dosing [16]. The current study is one of the first, to our knowledge, to pilot the implementation of PGx testing in a pediatric chronic pain clinic. Current PGx clinical guidelines exist for analgesics, such as ibuprofen, celecoxib, tramadol, and codeine, as well as commonly used antidepressants for pain. Results from the current preliminary pilot study indicate that children and adolescents, their caregivers, and pain clinic prescribers found PGx testing to be relevant, feasible, and acceptable, with PGx recommendations being implemented in all but one case, although no explanation was provided for the exception.

At study enrollment, over half the study sample were taking medications (including analgesics and psychotropic medications) that carry clinical PGx guidelines. Over 20 percent of participants who were taking medication at the time of receiving their PGx test results had an actionable recommendation for non-standard dosing to their current pharmacotherapy based on PGx testing. Overall, based on the genotyping of drug-metabolizing genes, half of all study participants were predicted to benefit from non-standard dosing for pain and psychotropic pharmacotherapy, including NSAIDs, certain opioids, SSRIs, and SNRIs, that carry clinical PGx guidelines.

Studies investigating PGx for children and adolescents with chronic pain have been very limited. One study assessing the influence of PGx testing on pain intensity and ADRs was conducted on pediatric patients seen in the emergency department for acute fracture [35]. Results indicated that *CYP2C9* * 2 was associated with fewer ADRs to ibuprofen compared to those with a normal functioning allele but did not impact clinical effectiveness or ADRs associated with oxycodone use. Authors cautioned that their sample was small and that metabolism of drugs in children and adolescents may differ from adults. In another study, 19 pediatric patients with chronic pain were referred for PGx testing due to analgesic ineffectiveness. Results showed a high incidence (16; 84%) of *CYP2D6* genetic variation among these children [36].

### 4.1. Clinical Considerations

Taken together, there is potential to predict certain patient outcomes and responses to analgesic and psychotropic pharmacotherapy. In our cohort, the results of four participants warrant attention: participants A, B, E, and G.

Variation in CYP2C19 metabolism may have influenced the responses of participants A and G to sertraline and escitalopram, respectively. According to CPIC guidelines, an increase in metabolism of escitalopram to its less active compounds in individuals with rapid or ultra-rapid CYP2C19 metabolism compared to those with normal CYP2C19 metabolism decreases the likelihood of clinical benefit due to lower plasma levels [32]. Conversely, CYP2C19 normal metabolizers are generally expected to respond well to standard dosing. The CYP2C19 metabolizer status observed in these two participants aligns with their reported responses. Similarly, the frequency of different CYP2C19 metabolizer statuses varies globally and depends on genetic ancestry. CYP2C19 rapid metabolizers are found in 2 to 27% of the global population, with the lowest prevalence observed in Oceanic ancestry. In contrast, CYP2C19 poor metabolizers can be as high as 70% in certain Pacific Islander populations but generally range between 2 and 15% worldwide [16].

Participants B and E reported having taken codeine, although this drug is generally not recommended for use in pediatric patients, especially those under 12 years of age [37]. Codeine is an opioid analgesic that requires metabolic activation by the CYP2D6 enzyme to exert its full analgesic effect by converting codeine into its active metabolite, morphine. The metabolism of codeine exhibits substantial interindividual variability due to the highly polymorphic nature of the *CYP2D6* gene, with genetic variation resulting in different metabolizer phenotypes: poor metabolizers, intermediate metabolizers, normal metabolizers, and ultra-rapid metabolizers [34,38]. Participant B had been categorized as a CYP2D6 poor metabolizer and is predicted to possess little to no functional CYP2D6 enzyme activity. Consequently, CYP2D6 poor metabolizers could experience insufficient pain relief from codeine as they lack the ability to produce morphine from codeine, as corroborated by participant B’s reported lack of response. It is important to note that CYP2D6 poor metabolism is observed in 2 to 6% of the global population, with the frequency varying based on genetic ancestry [39]. On the other hand, CYP2D6 ultra-rapid metabolizers carry more than two normal-function copies of the *CYP2D6* gene, leading to increased enzyme activity and accelerated conversion of codeine to morphine [34]. This can result in higher levels of morphine in the bloodstream, which may lead to symptoms of morphine overdose, including extreme drowsiness, confusion, and shallow breathing, and in some severe instances can be fatal. Indeed, participant E, who was an ultra-rapid CYP2D6 metabolizer, indicated that they had an enhanced analgesic response to codeine but felt drowsy. Ultra-rapid CYP2D6 metabolism is present in 1 to 11% of the global population, with the frequency also varying across various genetic ancestries [16].

Families were more likely to enroll in PGx testing at their POC visit (reactive testing) compared to receiving PGx testing while on a multi-month waitlist for an initial intake in the chronic pain clinic (preemptive testing). While preemptive PGx testing may be most useful to assist prescribers in tailoring pharmacotherapy from the outset of care (versus reactive testing), it may be that families have more readiness to pursue PGx testing following a comprehensive pain assessment and obtaining a pain diagnosis and education about chronic pain from a specialist. Given the benefit of prescribers receiving PGx recommendations prior to making medication and prescribing choices, future efforts should be directed at better understanding the perspectives of children and their caregivers on any barriers to receiving PGx testing while on a waitlist and any factors that could increase readiness to uptake PGx testing prior to a POC visit.

The fact that receiving PGx testing and recommendations improved children and their caregivers’ confidence and willingness to take medications merits mention. While analgesics are cited by children with pain and their caregivers as the top strategy to manage pain as compared to non-pharmacological strategies [40], multiple studies document caregivers’ hesitancy to use analgesics due to fear of adverse effects and addiction [41,42]. Additionally, many children and adolescents referred to chronic pain clinics have dealt with their pain for years and have tried a number of analgesics with unsuccessful effect. Anecdotally, these children and their caregivers often express limited hope that medications will have a positive impact on their symptoms and worry about ADRs, which can contribute to poor adherence to medication regimens, ultimately undermining medication effectiveness. Since PGx testing provides individualized recommendations for medications based on a patient’s genetic make-up, this may reduce hesitancy to administer analgesics based on fears of ADRs and improve trust in a medication’s effectiveness. The current study’s finding that PGx testing enhances child and caregiver attitudes towards taking medications should be further assessed to determine whether PGx testing influences behaviors such as adherence to medication regimens, particularly recognizing the importance of positive expectations in influencing medication effects [43].

### 4.2. Study Limitations

Prior studies examining PGx testing for pain management, conducted primarily in adults, showed promising genotype-guided therapy for opioid use compared to standard prescribing [21,44]; however, this was not the scope of our research. Our study focused on genes that have clinically actionable PGx guidelines available. By identifying CYP2C9, CYP2D6, and CYP2C19 enzyme function, HCPs can better predict an individual’s responses to various analgesics, thus optimizing dosing and mitigating the risk of ADRs, drug toxicity, or non-response. While the aforementioned metabolism genes offer an accessible entry point for understanding the activation or breakdown of medications used in pain management and mental health, further research is essential to understand and investigate broader impact, considering that certain patients benefit more than others.

This is the first pilot study to explore the use of PGx testing in pediatric chronic pain, highlighting the exploratory nature of the work. Our study population was limited to pediatric patients experiencing chronic pain, and the relatively small sample size limited our ability to explore the influence of non-pharmacological treatment factors and pain characteristics in association with PGx results, which may further reduce the generalizability of our findings to broader patient populations. While prior studies have largely focused on adult populations, direct comparisons were beyond the scope of this work; however, future research may benefit from exploring parallels between pediatric and adult PGx applications. Additionally, the study design was non-randomized and relied on convenience sampling, which should be acknowledged as a methodological limitation. There is significant inter-patient variability in clinical outcomes based on different drug–gene interactions and disease states, with minimal PGx endorsement for pediatric mental health indications [45,46]. This could be due to the multiple enzyme pathways and pharmacodynamic factors of commonly prescribed analgesics or psychiatric medications [47]. Certain patients, including those treated with medications primarily influenced by the activity of one enzyme, such as proton pump inhibitors or immunosuppressants, represent the low-hanging fruit of successful PGx implementation given their high level of clinical evidence [32,48]. These drug–gene interactions highlight the most direct targets for PGx-guided medication prescribing and thus can be more easily implemented than others. A further limitation of PGx testing for individuals with chronic pain rests in the fact that some medications frequently used to treat pain, including gabapentin, pregabalin, morphine, and hydromorphone, do not yet carry validated PGx guidelines, limiting the ability to inform prescribing decisions. Finally, we were not able to interrogate pharmacodynamic genes that code for drug targets such as ion channels, receptors, and transporters that are crucial to understanding pain as well as pain relief and may have an impact on effective medication management.

## 5. Conclusions

Pediatric patients in the mental health and pain management fields may benefit significantly from the application of PGx-guided prescribing, specifically regarding genes that influence drug metabolism. Indeed, based on the genotyping of drug-metabolizing genes, half of our participants were predicted to benefit from non-standard dosing of pain and psychotropic medications that carry clinical PGx guidelines. Variations in these metabolism genes can affect how individuals process medications, leading to differences in drug efficacy and the risk of side effects. The complexity of pain and mental health conditions, which are influenced by a wide range of biological, psychological, and environmental factors, and the interactions between these different pathways, complicate treatment responses. While PGx-guided prescribing received high satisfaction ratings from study participants, their caregivers, and their prescribers, and while it can provide insights into how certain medications may be processed by specific enzymes, it does not account for the full spectrum of factors influencing treatment outcomes. As a result, the predictive power of this testing is only one piece of a much larger puzzle in individualized care for these conditions. Further research using larger samples should focus on interrogating additional genetic variants with pharmacodynamic associations. This may increase the possibility of identifying more nuanced drug–gene interactions and variations impacting patient responses to pain medications.

## Figures and Tables

**Table 1:** Participant demographics.

Demographics	N = 46
**Age (years)**	
Mean (SD)	14.5 (2.28)
[Range]	[8, 17]
**Sex**	**n (%)**
Female	35 (76.1)
Male	11 (23.9)
**Pain Duration (years)**	
Mean (SD)	3.58 (3.24)
[Range]	[0.5, 13]
Type of Chronic Pain *	**n (%)**
Musculoskeletal	33 (71.7)
Headache	9 (19.6)
Abdominal	8 (17.4)
Neuropathic	4 (8.7)
Pelvic	2 (4.3)
Psychological Diagnoses *	**n (%)**
Anxiety disorder	28 (60.9)
ADHD	11 (23.9)
Intellectual/learning disability	10 (21.7)
Depression	7 (15.2)
Autism spectrum disorder	5 (10.9)
Tourette syndrome	2 (4.3)
Somatoform disorder	2 (4.3)
Other (PTSD, psychotic disorder)	2 (4.3)
Other Concurrent Diagnoses *	
Orthopedic	8 (17.4)
Cardiac	6 (13.0)
Neurological	6 (13.0)
Gastrointestinal	6 (13.0)
Musculoskeletal/Connective tissue	5 (10.9)
Other	8 (17.3)
**Baseline Scores at CPC Intake †**	**Mean (SD)**
PROMIS Pain Interference	65.87 (7.56)
PROMIS Anxiety	60.46 (8.77)
PROMIS Depressive Symptoms	58.16 (10.78)
PROMIS Mobility	36.09 (7.25)
Pain Catastrophizing	
Patient	64.28 (12.06)
Caregiver	61.18 (12.34)

* Chronic pain, psychological, and other concurrent diagnoses percentages are greater than 100% because several participants met diagnostic criteria for multiple diagnoses. Additionally, pain diagnoses were made by a pain physician based on clinical assessment at the pain clinic intake appointment and documented in the clinical assessment letter located in the medical record. Psychological diagnoses were documented in the psychologist’s note in the medical record. Psychological diagnoses were provided or confirmed by the psychologist as part of a psychological diagnostic assessment. † Baseline scores are based on self-report and represented as *t*-scores (mean = 50, SD = 10). Higher scores represent more of the domain being measured. SD, standard deviation; ADHD, attention-deficit/hyperactivity disorder; PTSD, post-traumatic stress disorder; CPC, chronic pain clinic; PROMIS, Patient-Reported Outcomes Measurement Information System.

**Table 2:** Analgesic and psychotropic medication usage across cohort.

Medication Use at Study Enrollment *	N (%)	PGx-Listed Medication Use at Time of PGx Report †	N (%)
Taking medication(s) as part of treatment 1 medication ≥2 medications	29/46 (63.0) 15/29 (51.7) 14/29 (48.2)	Taking PGx medication(s) as part of treatment 1 medication ≥2 medications	24/46 (52.2) 18/24 (75.0) 6/24 (25.0)
Taking analgesic NSAIDs ○ Ibuprofen ○ Celecoxib ○ Naproxen ○ Diclofenac Opioids ○ Morphine ○ Hydromorphone Anticonvulsants ○ Gabapentin ○ Pregabalin Acetaminophen Antihypertensive ○ Clonidine	25/29 (86.2) 15/25 (60.0) 5/15 (33.3) 2/15 (13.3) 5/15 (33.3) 3/15 (20.0) 2/25 (8.0) 1/2 (50.0) 1/2 (50.0) 7/25 (28.0) 6/7 (85.7) 1/7 (14.3) 6/25 (24.0) 1/25 (4.0)	Taking PGx analgesic NSAIDs ○ Ibuprofen ○ Celecoxib Opioids ○ Codeine ^‡^	13/24 (54.2) 12/13 (92.3) 9/13 (69.2) 3/13 (23.1) 1/13 (7.7)
Taking psychotropic SSRIs ○ Escitalopram ○ Sertraline ○ Fluoxetine ○ Citalopram SNRIs ○ Duloxetine ○ Venlafaxine TCAs ○ Amitriptyline	13/29 (44.8) 11/13 (84.6) 4/11 (36.4) 4/11 (36.4) 2/11 (18.2) 1/11 (9.1) 2/13 (14.4) 1/2 (50.0) 1/2 (50.0) 1/13 (7.7)	Taking psychotropic SSRIs ○ Sertraline ○ Escitalopram ○ Citalopram Anti-psychotics ○ Aripiprazole SNRIs ○ Venlafaxine Benzodiazepines ○ Clobazam Other PGx medications Hormonal contraceptives Antiemetics ○ Ondansetron ○ Metoclopramide PPIs ○ Omeprazole Beta-blockers ○ Metoprolol	12/24 (50.0) 9/24 (37.5) 4/9 (44.4) 4/9 (44.4) 1/9 (11.1) 1/24 (4.2) 1/24 (4.2) 1/24 (4.2) 4/24 (16.7) 2/24 (8.3) 1/2 (50.0) 1/2 (50.0) 2/24 (8.3) 1/24 (4.2)

* This includes medications that do not carry published clinical PGx practice guidelines. † This only includes medications that carry published clinical PGx practice guidelines that were listed in the PGx report. ‡ This participant was prescribed codeine as an adolescent at the time of participation in this study. SSRIs, selective serotonin reuptake inhibitors; SNRIs, serotonin–norepinephrine reuptake inhibitors; TCAs, tricyclic antidepressants; and PPIs, proton pump inhibitors.

**Table 3:** Timing of PGx results and consultation relative to POC appointment by study group.

Study Group	PGx Report Uploaded to Medical Chart Prior to POC Visit	Had PGx Consultation Prior to POC Visit
Cohort Waitlist/Preemptive testing Referral/Reactive testing	38/46 (82.6%)	33/46 (71.7%)
	23/31 (74.2%)	18/31 (58.1%)
	15/15 (100%)	15/15 (100%)

POC, point of care.

**Table 4:** Acceptability of PGx testing—patient and caregiver results.

Immediately After PGx Testing Consultation:	Agreed n (%)	
	Patients (N = 38) *	Caregivers (N = 40) *
I felt safe and supported throughout the PGx testing process.	38 (100)	40 (100)
The results from my/my child’s test were explained to me in an understandable way.	36 (94.7)	39 (97.5)
Being part of the PGx study helped me understand how I/my child would respond to the medications listed.	35 (92.1)	39 (97.5)
Being part of the PGx study reassured me that my/my child’s healthcare team is trying to pick the correct medication therapy for me.	36 (94.7)	38 (95)
PGx testing helped me understand myself/my child better.	35 (92.1)	38 (95)
PGx testing was fun for me to learn about.	36 (94.7)	40 (100)
PGx testing will help guide my/my child’s treatment decisions.	35 (92.1)	40 (100)
PGx testing helps my/my child’s physician make better decisions.	35 (92.1)	40 (100)
PGx testing is worth the waiting time.	36 (94.7)	39 (97.5)
PGx testing was not an invasion of my/my child’s privacy.	38 (100)	39 (97.5)
PGx testing reduced uncertainty about my/my child’s response to medications.	34 (89.5)	34 (85)
PGx testing increased my confidence in the effect of the medication for pain control.	31 (81.6)	38 (95)
**At 3-month follow-up**	**Patients** (N = 33) *	**Caregivers** (N = 33) *
Did having PGx testing increase your **confidence** in taking medication? Median, IQR †	7, 3	8, 5
Did having PGx testing improve your **willingness** to take medication more regularly (i.e., at the dosing schedule prescribed by your/your child’s physician)? Median, IQR †	5, 6	7, 6

* Completed questionnaires. † Scores indicated on a scale of 0 (absolutely no change in confidence/willingness in using these medications) to 10 (biggest increase in confidence/willingness ever in using these medications). IQR, interquartile range.

**Table 5:** Self-reported lifetime medication response and ADR predictions based on PGx results.

Participant	Medication	Side Effects	Symptom Change *	PGx Metabolizer Status	PGx Response/ADR Prediction
A	Sertraline	No	Better	CYP2C19 NM	Normal metabolism
	Ondansetron	No	Moderately better	CYP2D6 NM	Normal metabolism
B	Codeine	No	No change	CYP2D6 PM	Reduced conversion to morphine leading to reduced clinical effect (analgesia)
C	Pantoprazole	No	No change	CYP2C19 RM	Increased metabolism (decreased drug plasma levels) leading to reduced drug response and/or therapeutic failure
D	Lansoprazole	No	Little better	CYP2C19 IM	Decreased metabolism (increased drug plasma levels) leading to increased chance of efficacy
E	Codeine	Yes—drowsy	Moderately better	CYP2D6 UM	Increased metabolism to morphine leading to higher risk of side effects
F	Lansoprazole	No	Moderately better	CYP2C19 IM	Decreased metabolism (increased drug plasma levels) leading to increased chance of efficacy
G	Citalopram	No	Almost the same	CYP2C19 RM	Increased metabolism leading to decreased clinical benefit

NM, normal metabolizer; PM, poor metabolizer; IM, intermediate metabolizer; RM, rapid metabolizer; and UM, ultra-rapid metabolizer. * Symptom change derived from the Patient Global Impression of Change Scale: “Moderately better”—slight but noticeable change; “No change”—or function has worsened; “Little better”—but no noticeable change; and “Almost the same”—hardly any change at all.

## Data Availability

The datasets generated during and/or analyzed during the current study are available from the corresponding author upon reasonable request due to privacy and ethical reasons.

## Abbreviations

The following abbreviations are used in this manuscript:

PGx	Pharmacogenetics
ADRs	Adverse Drug Reactions
HCPs	Health Care Providers
CPC	Chronic Pain Clinic
EMR	Electronic Medical Record
POC	Point of Care

## Appendix A

**Table A1:** Medication Use History Questionnaire. To be administered to patient and caregiver (together, 1 questionnaire) immediately after consent is provided and before the PGx consultation.

**Has your child ever taken a pain medication before?** Y/N *If N, skip to 2.* (a) *If Y*, **please indicate which pain medications your child has taken:** - Celecoxib (Celebrex) - Codeine - Flurbiprofen (Ansaid, Ocufen, Strepfen) - Ibuprofen (Advil) - Meloxicam (Mobic, Metacam) - Oxycodone (Oxycontin) - Piroxicam (Feldene) - Tramadol (Ultram) - Amitriptyline (Elavil) - Desipramine (Norpramin) - Imipramine (Tofranil) - None of the above (b) [*for each medication checked above*] **Did your child experience any side effects from this medication? (check all that apply)** (a) *GI side effects (diarrhea, constipation, upset stomach, nausea, vomiting, ulcers)* (b) *Difficulties breathing* (c) *Bleeding* (d) *Drowsiness, dizziness* (e) *Others:* __________________________________ (c) [*for each medication checked above*] **Since beginning this medication, how would you describe the change in your child’s pain?** (a) *No change (or function has worsened)* (b) *Almost the same, hardly any change at all* (c) *A little better, but no noticeable change* (d) *Somewhat better, but the change has not made any real difference* (e) *Moderately better, and a slight but noticeable change* (f) *Better, and a definite improvement that has made a real and worthwhile difference* (g) *A great deal better, and a considerable improvement that has made all the difference* (d) [*for each medication checked above*] **Who prescribed this medication?** (a) *Family doctor* (b) *Pediatrician* (c) *ER doctor* (d) *Speciality appointment (i.e., rheumatology, pain, psychiatry, GI, etc.)* (e) *Other: _________* (e) [*for each medication checked above*] **Is your child still taking this medication?** Y/N *If N, **Why?*** (a) *This medication did not help* (b) *My child stopped this medication because of side effects* (c) *My child no longer needed this medication (i.e., pain subsided)* (d) *Other: ___________*
2. **Has your child ever been prescribed a psychiatric medication before?** Y/N *If N, skip to 3.* (a) *If Y*, **please indicate which psychiatric medications your child has been prescribed:** - Aripiprazole (Abilify) - Atomoxetine (Strattera) - Brexipiprazole (Rexulti) - Citalopram (Celexa) - Clobazam (Sympazan, Onfi) - Clomipramine (Anafranil) - Clozapine (Clozaril, FazaClo) - Doxepin (Silenor) - Escitalopram (Lexapro) - Fluvoxamine (Luvox) - Haloperidol (Haldol, Peridol) - Nortriptyline (Pamelor, Noritren) - Paroxetine (Paxil, Seroxat, Loxamine) - Perphenazine (Trilafon) - Pimozide (Orap) - Pitolisant (Wakix) - Risperidone (Risperdal) - Sertraline (Zoloft) - Thioridazine (Mellaril) - Trimipramine (Surmontil) - Venlafaxine (Effexor) - Vortioxetine (Trintellix) - Zuclopenthixol (Cisordinol, Clopixol) - None of the above (b) [*for each medication checked above*] **Did your child experience any side effects from this medication? (check all that apply)** (a) *GI side effects (diarrhea, constipation, upset stomach, nausea, vomiting, ulcers)* (b) *Difficulties breathing* (c) *Bleeding* (d) *Drowsiness, dizziness* (e) *Others: __________________________________* (c) [*for each medication checked above*] **Since beginning this medication, how would you describe the change in your child’s pain?** (a) *No change (or function has worsened)* (b) *Almost the same, hardly any change at all* (c) *A little better, but no noticeable change* (d) *Somewhat better, but the change has not made any real difference* (e) *Moderately better, and a slight but noticeable change* (f) *Better, and a definite improvement that has made a real and worthwhile difference* (g) *A great deal better, and a considerable improvement that has made all the difference* (d) [*for each medication checked above*] **Who prescribed this medication?** (a) *Family doctor* (b) *Pediatrician* (c) *ER doctor* (d) *Speciality appointment (i.e., rheumatology, pain, psychiatry, GI, etc.)* (e) *Other: _________* (e) [for each medication checked above] **Is your child still taking this medication?** Y/N *If N, **why?*** (a) *This medication did not help* (b) *My child stopped this medication because of side effects* (c) *My child no longer needed this medication (i.e., pain subsided)* (d) *Other: ___________*
3. **Has your child ever been prescribed a gastroenterology medication before?** Y/N (a) *If Y*, **please indicate which gastroenterology medications your child has been prescribed:** - Dexlansoprazole (Dexilant) - Dronabinol (Marinol) - Lansoprazole (Prevacid) - Omeprazole (Prilosec) - Ondansetron (Zofran, Zuplenz) - Pantoprazole (Protonix) - Metoclopramide (Reglan, Metozolv) - Tropisetron - None of the above (b) [*for each medication checked above*] **Did your child experience any side effects from this medication? (check all that apply)** (a) *GI side effects (diarrhea, constipation, upset stomach, nausea, vomiting, ulcers)* (b) *Difficulties breathing* (c) *Bleeding* (d) *Drowsiness, dizziness* (e) *Others: __________________________________* (c) [*for each medication checked above*] **Since beginning this medication, how would you describe the change in your child’s pain?** (a) *No change (or function has worsened)* (b) *Almost the same, hardly any change at all* (c) *A little better, but no noticeable change* (d) *Somewhat better, but the change has not made any real difference* (e) *Moderately better, and a slight but noticeable change* (f) *Better, and a definite improvement that has made a real and worthwhile difference* (g) *A great deal better, and a considerable improvement that has made all the difference* (d) [*for each medication checked above*] **Who prescribed this medication?** (a) *Family doctor* (b) *Pediatrician* (c) *ER doctor* (d) *Speciality appointment (i.e., rheumatology, pain, psychiatry, GI, etc.)* (e) *Other: _________*
4. [*for each medication checked above*] **Is your child still taking this medication?** Y/N **If N, why?** (a) *This medication did not help* (b) *My child stopped this medication because of side effects* (c) *My child no longer needed this medication (i.e., pain subsided)* (d) *Other: ___________*

## Appendix B

**Table A2:** PGx Testing Acceptability—Patient and Caregiver Versions. To be administered as separate weblinks for patients and caregivers via REDCap one day after PGx consultation.

Please indicate how the following statements apply to you:
*Completely Agree/Strongly Agree/Agree/Somewhat Agree/Somewhat Disagree/Disagree/Strongly Disagree/Completely Disagree* I felt safe and supported throughout the PGx testing process. The results from my/my child’s test were explained to me in an understandable way. Being part of the PGx study helped me understand how I/my child would respond to the medications listed. Being part of the PGx study reassured me that my/my child’s healthcare team is trying to pick the correct medication therapy for me.
5. Pharmacogenomics testing: *Completely Agree/Strongly Agree/Agree/Somewhat Agree/Somewhat Disagree/Disagree/Strongly Disagree/Completely Disagree* (a) Helped me understand myself/my child better (b) Was fun for me to learn about (c) Will help guide my/my child’s treatment decisions (d) Helps my/my child’s physician make better decisions (e) Is not worth the waiting time (f) Was an invasion of my/my child’s privacy (g) Reduced uncertainty about my/my child’s response to medications (h) Increased my confidence in the effect of the medication for pain control (i) *Other: ________________________________*
6. Please rate each part of the pharmacogenetics report: (a) Clear language (b) Logical layout/flow (c) Informative (d) Helpful (e) Easy to understand
7. **Open-ended question:** Is there any other feedback you have about your experience with pharmacogenomics testing?

## Appendix C

**Table A3:** PGx Testing Implementation/Acceptability Questionnaire. Questionnaire to be administered to HCPs through REDCap following a point-of-care appointment with each participant.

Chronic Pain Sample
**Did you prescribe a medication for this patient today or at a recent visit?** *Y/N + provide comment box*
2. **Were any of the following medications prescribed for this patient?** *Y/N + provide comment box* Pain (Neuropathic*) Celecoxib, Codeine, Flurbiprofen, Ibuprofen, Meloxicam, Oxycodone, Piroxicam, Tramadol, Amitriptyline *, Desipramine *, Imipramine* Psychiatry Aripiprazole, Atomoxetine, Brexipiprazole, Citalopram, Clobazam, Clomipramine, Clozapine, Doxepin, Escitalopram, Fluvoxamine, Haloperidol, Nortriptyline, Paroxetine, Perphenazine, Pimozide, Pitolisant, Risperidone, Sertraline, Thioridazine, Trimipramine, Venlafaxine, Vortioxetine, Zuclopenthixol Gastroenterology Dexlansoprazole, Dronabinol, Lansoprazole, Omeprazole, Ondansetron, Pantoprazole, Metoclopramide, Tropisetron *If response to 2. is YES:* **2a. If any medications on the PGx list were prescribed, did you implement the PGx test recommendation? (i.e., PGx recommendations may be to provide standard dosing; deviation from standard dosing; select a different medication)** *Y/N + provide comment box* *If response to 2. is NO:* **2b. If you did not prescribe a medication from the PGx list, did you find the PGx testing results to be helpful information for prescribing decisions? (i.e., future or past medication choices)** *Y/N + provide comment box*
3. **Did you feel PGx testing results were relevant for this patient?** *Y/N + provide comment box*

## Appendix D

**Table A4:** PGx Testing Follow-up Questionnaire. To be administered to both patient and caregiver (1 questionnaire link) via REDCap 3 months after point-of-care appointment.

**Did having PGx testing increase your confidence in taking medication?** *Please indicate below, 0 being absolutely no change in my confidence in using these medications and 10 being the biggest increase in confidence ever in using these medications.* Patient: _____ Parent: _____
2. **Did having PGx testing improve your willingness to take medication more regularly (i.e., at the dosing schedule prescribed by your child’s physician)?** *Please indicate below, 0 being absolutely no change in my willingness in using these medications and 10 being the biggest increase in willingness ever in using these medications.* Patient: _____ Parent: _____
3. **Were you prescribed any pain medications since you were enrolled in the study? Y/N.** *If N, skip to 4.* (a) *If Y,* **which medication?** Celecoxib (Celebrex) Codeine Flurbiprofen (Ansaid, Ocufen, Strepfen) Ibuprofen (Advil) Meloxicam (Mobic, Metacam) Oxycodone (Oxycontin) Piroxicam (Feldene) Tramadol (Ultram) Amitriptyline (Elavil) Desipramine (Norpramin) Imipramine (Tofranil) None of the above (b) [*for each medication checked above*] **Did your child experience any side effects from this medication? (check all that apply)** (a) GI side effects (diarrhea, constipation, upset stomach, nausea, vomiting, ulcers) (b) Difficulties breathing (c) Bleeding (d) *Drowsiness, dizziness* (e) *Others: __________________________________* (c) [*for each medication checked above*] **Since beginning this medication, how would you describe the change in your child’s pain?** (a) *No change (or function has worsened)* (b) *Almost the same, hardly any change at all* (c) *A little better, but no noticeable change* (d) *Somewhat better, but the change has not made any real difference* (e) *Moderately better, and a slight but noticeable change* (f) *Better, and a definite improvement that has made a real and worthwhile difference* (g) *A great deal better, and a considerable improvement that has made all the difference* (d) [*for each medication checked above*] **Who prescribed this medication?** (a) *Family doctor* (b) *Pediatrician* (c) *ER doctor* (d) *Speciality appointment (i.e., rheumatology, pain, psychiatry, GI, etc.)* (e) [*for each medication checked above*] **Did this doctor use the PGx results when prescribing this medication?** (a) *Yes, my doctor used the results* (b) *The results were available but my doctor wasn’t interested in looking at them* (c) *The results were available but the medication I was prescribed was not on the list* (d) *I/we had not received my PGx results at the time that I was prescribed medica-tion* (f) [*for each medication checked above*] **Is your child still taking this medication? Y/N.** *If N*, why? (a) *This medication did not help* (b) *My child stopped this medication because of side effects* (c) *My child no longer needed this medication (i.e., pain subsided)* (d) *Other: ___________*
4. **Has your child ever been prescribed a psychiatric medication before?** Y/N *If N, skip to 5.* (a) *If Y*, **please indicate which psychiatric medications your child has been prescribed:** - Aripiprazole (Abilify) - Atomoxetine (Strattera) - Brexipiprazole (Rexulti) - Citalopram (Celexa) - Clobazam (Sympazan, Onfi) - Clomipramine (Anafranil) - Clozapine (Clozaril, FazaClo) - Doxepin (Silenor) - Escitalopram (Lexapro) - Fluvoxamine (Luvox) - Haloperidol (Haldol, Peridol) - Nortriptyline (Pamelor, Noritren) - Paroxetine (Paxil, Seroxat, Loxamine) - Perphenazine (Trilafon) - Pimozide (Orap) - Pitolisant (Wakix) - Risperidone (Risperdal) - Sertraline (Zoloft) - Thioridazine (Mellaril) - Trimipramine (Surmontil) - Venlafaxine (Effexor) - Vortioxetine (Trintellix) - Zuclopenthixol (Cisordinol, Clopixol) - None of the above (b) [*for each medication checked above*] **Did your child experience any side effects from this medication? (check all that apply)** (a) *GI side effects (diarrhea, constipation, upset stomach, nausea, vomiting, ulcers)* (b) *Difficulties breathing* (c) *Bleeding* (d) *Drowsiness, dizziness* (e) *Others: __________________________________* (c) [*for each medication checked above*] **Since beginning this medication, how would you describe the change in your child’s pain?** (a) *No change (or function has worsened)* (b) *Almost the same, hardly any change at all* (c) *A little better, but no noticeable change* (d) *Somewhat better, but the change has not made any real difference* (e) *Moderately better, and a slight but noticeable change* (f) *Better, and a definite improvement that has made a real and worthwhile difference* (g) *A great deal better, and a considerable improvement that has made all the difference* (d) [*for each medication checked above*] **Who prescribed this medication?** (a) *Family doctor* (b) *Pediatrician* (c) *ER doctor* (d) *Speciality appointment (i.e., rheumatology, pain, psychiatry, GI, etc.)* (e) *Other: _________* (e) [*for each medication checked above*] **Did this doctor use the PGx results when prescribing this medication?** (a) *Yes, my doctor used the results* (b) *The results were available but my doctor wasn’t interested in looking at them* (c) *The results were available but the medication I was prescribed was not on the list* (d) *I/we had not received my PGx results at the time that I was prescribed medica-tion* (f) [*for each medication checked above*] **Is your child still taking this medication?** *Y/N* *If N, **why?*** (a) *This medication did not help* (b) *My child stopped this medication because of side effects* (c) *My child no longer needed this medication (i.e., pain subsided)* (d) *Other: ___________*
5. **Has your child ever been prescribed a gastroenterology medication before?** Y/N (a) *If Y*, **please indicate which gastroenterology medications your child has been prescribed:** - Dexlansoprazole (Dexilant) - Dronabinol (Marinol) - Lansoprazole (Prevacid) - Omeprazole (Prilosec) - Ondansetron (Zofran, Zuplenz) - Pantoprazole (Protonix) - Metoclopramide (Reglan, Metozolv) - Tropisetron - None of the above (b) [*for each medication checked above*] **Did your child experience any side effects from this medication? (check all that apply)** (a) *GI side effects (diarrhea, constipation, upset stomach, nausea, vomiting, ulcers)* (b) *Difficulties breathing* (c) *Bleeding* (d) *Drowsiness, dizziness* (e) *Others: __________________________________* (c) **[for each medication checked above] Since beginning this medication, how would you describe the change in your child’s pain?** (a) *No change (or function has worsened)* (b) *Almost the same, hardly any change at all* (c) *A little better, but no noticeable change* (d) *Somewhat better, but the change has not made any real difference* (e) *Moderately better, and a slight but noticeable change* (f) *Better, and a definite improvement that has made a real and worthwhile difference* (g) *A great deal better, and a considerable improvement that has made all the difference* (d) [*for each medication checked above*] **Who prescribed this medication?** (a) *Family doctor* (b) *Pediatrician* (c) *ER doctor* (d) *Speciality appointment (i.e., rheumatology, pain, psychiatry, GI, etc.)* (e) *Other: _________* (e) [*for each medication checked above*] **Did this doctor use the PGx results when prescribing this medication?** (a) *Yes, my doctor used the results* (b) *The results were available but my doctor wasn’t interested in looking at them* (c) *The results were available but the medication I was prescribed was not on the list* (d) *I/we had not received my PGx results at the time that I was prescribed medica-tion* (f) [*for each medication checked above*] **Is your child still taking this medication?** *Y/N* *If N, **why?*** (a) *This medication did not help* (b) *My child stopped this medication because of side effects* (c) *My child no longer needed this medication (i.e., pain subsided)* (d) *Other: ___________*

## Appendix E

**Table A5:** PGx Testing Overall Feedback Questionnaire. Questionnaire to be administered to all HCPs once participant recruitment has been completed.

Please indicate how the following statements apply to you:
*Completely Agree Strongly Agree/Agree/Somewhat Agree/Somewhat Disagree/Disagree/Strongly Disagree/Completely Disagree* I am notified which of my patients have received PGx testing. The method of notification is effective—i.e., email to provider within EPIC. The PGx results and report are easy to locate in the patient chart. The PGx report recommendations are easy to interpret and utilize for clinical decision-making. The PGx results are easy for me to explain to patients and parents. I found the PGx results helpful in making medication recommendations to my patients. I would consider requesting a PGx consult for future patients. Please note that the PGx report provides the following types of recommendations: (a) provide standard dosing; (b) consider a deviation from standard dosing; (c) consider a different medication. The following question refers to how often you implement a recommendation from the PGx test for your patients. We understand there are many medications that you might prescribe that are not listed or tested for, but we are interested in whether you implemented a PGx recommendation for those medications currently listed on the PGx test. When prescribing a medication for your patient that is on the PGx test (e.g., ibuprofen, celecoxib, amitriptyline, tramadol, oxycodone, other medications listed on the PGx that are relevant for your patient such as psychiatric medications), do you implement the PGx recommendation: (a) Always (b) Most of the time (c) Sometimes (d) Infrequently (e) Never PGx testing for pain medications is a new initiative. Therefore, we are interested in any feedback you can provide about your experience with PGx testing provided to your patients. Please provide any additional feedback here (any areas that you like, areas for improvement.) *comment box*

## Appendix F

**Table A6:** Alleles tested on the Agena MassARRAY^®^ platform used in this protocol. The functional status of each allele is indicated.

Gene	Predicted * Allele	Functional Status
*CYP2C9*	* 1	Normal Function
	* 2	Decreased Function
	* 3	No Function
	* 4	Decreased Function
	* 5	Decreased Function
	* 6	No Function
	* 8	Decreased Function
	* 11	Decreased Function
	* 12	Decreased Function
	* 13	No Function
	* 15	No Function
	* 25	No Function
	* 27	Uncertain Function
*CYP2C19*	* 1	Normal Function
	* 2	No Function
	* 3	No Function
	* 4A	No Function
	* 4B	No Function
	* 5	No Function
	* 6	No Function
	* 7	No Function
	* 8	No Function
	* 17	Increased Function
*CYP2D6* **	* 1	Normal
	* 2	Normal Function
	* 3	No Function
	* 4	No Function
	* 5	No Function
	* 6	No Function
	* 7	No Function
	* 8	No Function
	* 9	Decreased Function
	* 10	Decreased Function
	* 11	No Function
	* 12	No Function
	* 14A	Decreased Function
	* 14B	Decreased Function
	* 15	No Function
	* 17	Decreased Function
	* 18	No Function
	* 19	No Function
	* 20	No Function
	* 29	Decreased Function
	* 41	Decreased Function
	* 69	No Function

** CYP2D6 Copy Number Variant (CNV) analysis is performed using the Agena Veridose CYP2D6 CNV panel, which detects both CNVs and hybrid alleles and includes 22 assays to interrogate seven regions (5′, exon 1, intron 2, intron 4, intron 6, intron 7, and exon 9) of the CYP2D6 gene.
